# Pediatric Intensive Care Unit Conflict Management Perspectives Among Physician and Nurse Leaders

**DOI:** 10.1001/jamanetworkopen.2025.9783

**Published:** 2025-05-15

**Authors:** Aleksandra E. Olszewski, Seema K. Shah, Leonardo Barrera, Leopoldo Castillo, Irini Kolaitis, Denise M. Goodman, Erin Paquette

**Affiliations:** 1 Department of Pediatric Critical Care Medicine, University of Pittsburgh, Pittsburgh, Pennsylvania; 2 Department of Pediatrics, Northwestern University Feinberg School of Medicine, Chicago, Illinois; 3 Stanley Manne Children’s Research Institute, Ann & Robert H. Lurie Children’s Hospital of Chicago, Chicago, Illinois; 4 Division of Hospital-Based Medicine, Ann and Robert H. Lurie Children’s Hospital of Chicago, Chicago, Illinois; 5 Division of Critical Care Medicine, Ann and Robert H. Lurie Children’s Hospital of Chicago, Chicago, Illinois

## Abstract

**Importance:**

Decision-making conflict is common in the pediatric intensive care unit (PICU) and associated with negative outcomes for patients, families, and teams. Disparities in conflict outcomes are reported, yet no studies have explored conflict management approaches.

**Objective:**

To understand approaches to conflict mediation and escalation in the PICU.

**Design, Setting, and Participants:**

This national, multicenter, prospective, mixed-methods survey study recruited PICU physician and nursing directors from February to April 2023.

**Exposure:**

PICU size, conflict policy, behavior contract tracking.

**Main Outcomes and Measures:**

Closed- and open-ended survey questions were used to collect information on hospital policies, general conflict approaches, and specific approaches to scenarios.

**Results:**

The overall response rate was 57% (68 of 120 surveys, with 60 complete enough for analysis). Overall, 30 of 51 respondents (59%) identified as female, with a wide distribution of reported years in current role and percentage of time spent in clinical care. Institution regions varied, with an even distribution among institutions with different PICU sizes. Conflict strategies were used variably across institutions. Approximately 65% (32 of 49) reported policies for conflict management. Only 23% (10 of 43) tracked conflicts and their outcomes. Few institutions trained staff in conflict management techniques. Compared with institutions that did not track behavior contracts, those that did were more likely to call ethics consults for conflict management (3 of 12 [25%] vs 0 of 20; *P* = .04) and to implement behavior contracts for more objective reasons (8 of 11 [73%] vs 4 of 16 [25%]; *P* = .02). Leaders offered ideas for multipronged conflict prevention and response based on strategies implemented at their institutions but also highlighted needs and concerns with existing approaches.

**Conclusions and Relevance:**

In this mixed-methods survey study of PICU physician and nurse leaders, tracking and internal reporting of conflicts and outcomes were associated with more objectively applied interventions. The wide-ranging approaches and thresholds for escalation voiced by our respondents indicate a need to develop standardized and evidence-based processes to ensure greater effectiveness by clinical teams and leaders in addressing conflict and reduce potential disparities in outcomes. Respondents shared ideas for preventive and responsive processes that could be implemented and tested in the future. Learning from existing management approaches may help develop standardized, generalizable interventions to reduce conflict, improve interventions, and reduce subjectivity in the application of interventions.

## Key Points

**Question:**

How is decisional conflict between families and clinical teams managed?

**Findings:**

In this survey study of 60 pediatric intensive care unit (PICU) physician and nurse leaders, there was wide variability in approaches and opinions about best practices for conflict prevention, mediation, and management.

**Meaning:**

Our findings indicate a need to develop standardized, multipronged, and evidence-based processes to prevent and address conflict between clinical teams and families.

## Teaser

This survey study among physician and nurse leaders in pediatric intensive care units (PICUs) across the United States examines how decisional conflict between families and clinical care teams is handled.

## Introduction

Decision-making conflict in the pediatric intensive care unit (PICU) occurs when there is a dispute, disagreement, or difference of opinion related to the management of a patient that requires decision or action.^1,2,3^ The PICU involves urgent, high-stakes decision-making for the patients with the most severe illnesses in the hospital, requiring complex interpersonal communication in an environment that can be challenging and emotionally distressing for families and clinical teams.^2,3,4,5^ Unsurprisingly, decision-making conflict between patient families and clinical teams in the PICU is common, affecting 33% of all patients and as many as 80% of patients at the end of life.^2,3^ While some decision-making conflicts are unavoidable, others may be unnecessary and detrimental.^6^ Some PICU conflicts result in worse patient and family satisfaction and safety, team well-being, and family-centered care.^2,3,4,6^

Existing data demonstrate disparities in conflict occurrence.^2,3,7,8,9,10^ Certain patient populations are at increased risk of conflict, including those who belong to minoritized racial and ethnic groups, those who receive care in a language other than English, those with prolonged stays, those facing end-of-life decisions, and those with public insurance.^2,3,9^ Patient families at increased risk of conflict with clinical teams also may be at increased risk of more punitive, burdensome approaches when conflict does occur. For example, compared with White families, Black families are more likely to receive behavior contracts, security consults, and ethics consults.^8,9,10^

Effective conflict prevention and response could reduce the risks of occurrence, improve outcomes, and mitigate disparities in outcomes. Yet approaches to conflict management in the PICU remain underexplored.^6^ While some studies have qualitatively described conflict typology,^2,3,6,11^ to our knowledge, no studies have evaluated existing management approaches. In this prospective mixed-methods survey study of PICU physician and nurse leaders, we sought to understand existing approaches to conflict mediation, escalation, and family limitation. Given the paucity of prior work in this area, this was a descriptive study. Despite the study’s exploratory nature, we hypothesized that institutions overall were unlikely to have formal systematic monitoring and mediation approaches in place and that, if such approaches were in place, they were more likely found in larger PICUs.

## Methods

This study was deemed exempt by the Ann & Robert H. Lurie Children's Hospital of Chicago institutional review board. We informed respondents that survey completion was voluntary, and completion indicated consent. Survey design, distribution, and reporting adhered to the CHERRIES checklist.^12^ Reporting of qualitative analyses adhered to the Consolidated Criteria for Reporting Qualitative Research (COREQ) reporting guideline,^13,14^ and reporting of survey analyses adhered to the American Association for Public Opinion Research (AAPOR) reporting guideline.

### Identification and Contact of Survey Respondents

We used purposive and snowball sampling to identify key informants. Physician leaders from a diverse range of PICU sizes, locations, and types were identified using lists from Electronic Residency Application Service, the Children’s Hospital Association, and previously published data on PICUs in the United States.^15,16,17^ Physician leader identification and contact information was obtained via hospital webpages, key informants, and phone calls to PICUs. In the first survey distribution round, we asked physician leaders to identify names and email addresses for nursing leadership, to whom surveys were subsequently sent. The survey was open from February to April 2023.

### Survey Development

The survey (eAppendix in Supplement 1) was designed with multidisciplinary input from nursing, social work, ethics, and physician leadership across hospital divisions. Some questions were adapted from a survey tool on hospital safety and security, and others used previously published PICU conflict analyses.^3,6,18^ The survey contained general questions regarding conflict response, followed by 4 case-based scenarios, with both open- and closed-ended questions. The survey was validated through interviews and pilot testing with 8 physician leaders to improve the content, flow, and technical functionality.

### Survey Data Collection

We emailed 120 closed survey invitations with information sheets. We used Qualtrics (January 2023) web-based platform, with 1 question per page. Each respondent had a unique link that could be reviewed prior to submission but could only be completed once. If a survey was not completed, we sent as many as 2 follow-up emails.

### Qualitative Analysis

For the open-ended survey questions, we used qualitative description and content analysis.^19,20^ The codebook was developed iteratively by two authors (A.E.O. and L.C.). Codes were developed both inductively and deductively. A.E.O. and L.C. separately reviewed the summative answers to each open-ended question and independently developed draft codebooks. These draft codebooks were reviewed and revised by the research team, iteratively combining them into a single harmonized codebook, which A.E.O. and L.C. separately applied to the responses for answers for 3 of the survey questions. Then, A.E.O. and L.C. met to review codes and findings, generating a final codebook. A.E.O. and L.C. each independently coded every transcript. Both coders met to resolve any discrepancies until agreement was reached. Dedoose software version 9.0.17 was used. We organized the findings into several key themes, interpreted with reference to relevant literature. Survey participants did not provide feedback on study findings.

### Statistical Analysis

Univariate descriptive statistics were conducted for demographic variables, policy implementation, and conflict approaches. χ^2^ and Fisher exact tests were performed to test for differences between independent variables of PICU size (small [≤20 beds], medium [21-30 beds], or large [≥31 beds]) and policy implementation and tracking (present or absent), with dependent variables of conflict mediation techniques, common reasons for escalation, interventions for imposing limits, and reasons for behavior contract implementation. Statistical significance was defined as a 2-tailed *P* value less than .05. SPSS Statistics for Windows version 28.0 (IBM Corp) was used.

## Results

### Quantitative Results

Overall response rate was 57% (68 of 120), with 50% of surveys (60) complete enough for analysis (30% or greater completion) (Figure). Overall, 30 of 51 respondents (59%) identified as female, with a wide distribution of reported years in current role and percentage of time spent in clinical care (Table 1). Institution regions varied, with an even distribution among institutions with different PICU sizes.

**Figure.**
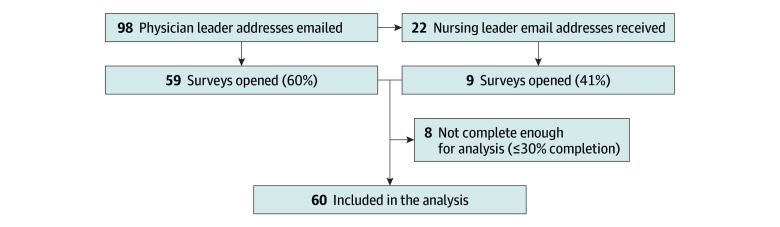
Flowchart Illustrating Study Recruitment

**Table 1.** Respondent and Institutional Characteristics^a^

Characteristic	No. (%)
**Respondents (n = 51)**	
Age, y	
26-35	1 (2)
36-45	19 (37)
46-55	20 (39)
>55	11 (22)
Gender	
Male	21 (41)
Female	30 (59)
Years in role	
<5	23 (45)
6-10	7 (14)
11-15	5 (10)
≥16	16 (31)
Time spent in patient care, %	
<25	7 (14)
25-50	18 (35)
51-75	7 (14)
>75	19 (37)
**Institutions (n = 52)**	
Region	
Midwest	16 (31)
Northeast	19 (37)
South	9 (17)
West	8 (15)
Location (n = 50)	
Urban	47 (94)
Rural	3 (6)
PICU beds, No.	
≤20	18 (34)
21-30	17 (33)
≥31	17 33)

Abbreviation: PICU, pediatric intensive care unit.

^a^ Institutional data includes both nurse and physician leadership responses, so the 51 to 52 responses summarized in the table may reflect a smaller total number of institutions.

Approaches to support systematic conflict monitoring and response varied (Table 2). Approximately 65% (32 of 49) reported conflict policies, 23% (10 of 43) tracked conflict outcomes, and 38% (12 of 32) tracked behavior contracts. Of these, 25% (3 of 12) tracked disparities in behavior contract use. While nearly all respondents (56 of 57 [98%]) had a diversity, equity, and inclusion (DEI) office, only 5% (2 of 40) systematically involved them in conflict management, with 52% (21 of 40) involving them in an ad hoc fashion. Respondents used the following interventions frequently (compared with sometimes or never): involvement of consultants (social work, 97% [57 of 59]; security, 19% [11]; ethics, 8% [5]), patient-family relations (56% [33]), and behavior contracts (32% [19]) (eFigure 1 in Supplement 1).

**Table 2.** Utilization of Institutional and Systematic Approaches for Conflict Monitoring and Response^a^

Approaches supporting systematic conflict monitoring and response	Reported use, No./ total No. (%)				*P* value
	Total responses	PICU size			
		≤20 Beds	21-30 Beds	≥31 Beds	
Policies for conflict management	32/49 (65)	10/13 (77)	6/14 (43)	10/13 (77)	.09
Tracking significant conflicts and outcomes	10/43 (23)	1/13 (8)	3/12 (25)	3/8 (36)	.22
Tracking behavior contract use	12/32 (38)	3/11 (27)	5/11 (45)	2/5 (40)	.67
Collaboration with DEI offices or similar institutions on conflict management					
Any	23/40 (57)	9/12 (75)	4/12 (33)	9/12 (75)	.05
Systematic	2/40 (5)	NA	NA	NA	NA
Ad hoc	21/40 (52)	NA	NA	NA	NA

Abbreviations: DEI, diversity, equity, and inclusion; NA, not applicable; PICU, pediatric intensive care unit.

^a^ Not all respondents filled out all portions of the survey, so the total number of responses by PICU bed size may not add up to the total number of responses per question.

Of conflict mediation techniques, 75% of respondents (42 of 56) indicated that they used deescalation frequently, 13% (7 of 55) used motivational interviewing frequently, and 20% (11 of 54) used third-party mediation frequently. Few staff received formal training in these techniques, with nursing staff most likely to receive it (Table 3).

**Table 3.** Reported Use of Mediation Techniques and Training in Each Technique for Different Care Team Members

Mediation technique and training	Respondents, No. (%)		
	Deescalation	Motivational interviews	Third-party mediation
Use of technique frequently or sometimes (vs never), No./total No. (%)	55/56 (98)	35/55 (64)	49/54 (91)
Training in technique by profession (n = 56)			
Bedside nursing	30 (53)	1 (2)	8 (14)
Nursing leadership	37 (66)	5 (9)	16 (29)
Medical trainees	15 (27)	4 (7)	4 (7)
Frontline clinicians	19 (34)	3 (5)	7 (13)
Attending staff	21 (38)	7 (13)	10 (18)
Medical leadership	23 (41)	5 (9)	15 (27)
Social work	30 (53)	8 (14)	13 (23)
Chaplains	20 (36)	4 (7)	8 (14)

Behavior contracts were used for a variety of indications (eFigure 2 in Supplement 1). Compared with institutions that did not track behavior contracts, those that did were more likely to call ethics consults for conflict management (3 of 12 [25%] vs 0 of 20; *P* = .04) and more likely to implement behavior contracts for physical and verbal aggression (8 of 11 [73%] vs 4 of 16 [25%]; *P* = .02), verbal aggression alone (9 of 11 [82%] vs 6 of 16 [37%]; *P* = .05), domestic violence or disturbance (5 of 11 [45%] vs 1 of 16 [6%]; *P* = .03), or nonadherence to hospital policies (4 of 11 [36%] vs 0 of 16; *P* = .02). We found no significant differences across hospital demographics (size, location, type) in terms of whether respondents identified that they had a policy for conflict, or tracking of behavior contracts.

### Qualitative Results

#### Existing Approaches to Prevent and Respond to Conflict

##### Preventive Approaches

Most respondents described the importance of conflict prevention, including through standardized proactive discussions, such as regular rounds with social work, ethics, and division leadership, where difficult cases, conflicts, and challenges are brought up by the clinical teams (Table 4). Respondents from one institution described PEACE (Pediatric Ethics and Communication Excellence) weekly rounds, intended to proactively identify problems early on and address them.^21^ A commonly described preventive approach was to set expectations, ideally as early as on admission, through care pledges, standard scripts on rounds, and posters with bidirectional expectations. Some institutions offered regularly scheduled care conferences for all patients that met certain criteria. Finally, a few respondents described that standard multidisciplinary decision support tools for common decisions served to prevent conflict. However, several respondents also said that decision support tools are needed but have not yet been created for many common decisions families face.

**Table 4.** Approaches Used to Prevent Conflict and Respond to Conflict^a^

Approach	Example	Representative quotation
**Preventive approaches**		
Regularly and proactively scheduled care conferences	NA	“Weekly care conferences can be used to help provided a more comprehensive understanding to the family.”
Standard multidisciplinary decision support tools	Tracheostomy decision-making pathway	“We… have a pathway for tracheostomy evaluation that is standard in our patients.…We would meet with the members of the trach[eostomy] evaluation team which include the ENT service, our home vent team, social work, palliative care, discharge planning team.”
Proactive clinical team discussions (regular social work, ethics, leadership rounds)	PEACE weekly rounds	“Long stay PICU patients as well as patients with repeat admissions are discussed. Conflicts, whether between medical team and patient family or between different medical teams, are identified and discussed. Medical care conferences are scheduled based on these discussions in efforts to resolve the conflict. Should this not alleviate the situation, there is a formal dispute resolution process that is followed.”
Expectation setting	Care pledges, family posters	“Care pledges… are posted throughout the hospital, and if families breech the guidelines of the care pledge, they are removed from the hospital. A ‘threat assessment’ team then determines when family can return.”
Trainings	Deescalation, conflict resolution	“We have gone through Welle training to assist in conflict resolution/deescalation.”
**Responsive approaches**		
Ad hoc care conferences, conversations at bedside	NA	“There is no formal process. We take a simple approach, and talk it out at the bedside like professionals.”
Multidisciplinary consults (palliative care, ethics, social work)	Ad hoc	“When words like ‘futility’ are used and there is disagreement between family and medical team, we often utilize our ethics team to help us unpack all the issues at play.”
	Formalized	“Social Work Intervention with Families and Teams (SWIFT), a team accessed via hospital operator for early de-escalation responses.”
		“Behavioral Emergency Response Team (BERT), a multidisciplinary team trained to respond, assess and intervene as needed for behavioral health emergencies in the inpatient setting.”
Escalation consults	Risk management, security, child protective services, legal services	“We may involve members of our Patient Relations and Legal teams. When behavior is outside acceptable norms, our nursing director, social work, and patient relations leaders typically meet with the family to discuss what behavior is outside the acceptable range and that the person exhibiting that behavior will be removed from the hospital if it continues.”
Behavior contracts	NA	“We have multiple different techniques depending on the conflict to address the issues. We use frequent family conferences to address communication issues, social work involvement and behavior contracts when policies are being broken repeatedly, security involvement and removal from premises if violent behavior or illegal behavior is identified, ethics involvement when inappropriate care is being requested, etc.”

Abbreviations: ENT, ear, nose, and throat; NA, not applicable; PEACE, Pediatric Ethics and Communication Excellence; PICU, pediatric intensive care unit.

^a^ These overall approaches to conflict were gleaned both from responses to questions about general approaches and from responses to questions about specific case-based conflicts. Detailed analysis of case responses is beyond the scope of this manuscript and will be included in a separate report.

##### Responsive Approaches

Responsive approaches that react to conflict between families and teams included care conferences and smaller discussions at the bedside (Table 4). Some respondents advocated that approaches were best done ad hoc to address important nuances. For example, one medical director (respondent 22) shared that there is no formal process at their institution, and that instead, “we talk it out like professionals.” Others shared frustrations with not having structured processes, as this was thought to lead to conflicts being “approached informally”; specifically, one nursing director (respondent 21) felt informal approaches typically translated to “following one heavy-weighted attending’s recommendations.” Many respondents shared that multidisciplinary consultation was useful with ethics, social work, and palliative care services. As conflicts escalated and became intractable, many respondents described utilizing legal services, patient relations, nursing management, risk management, security teams, child protective services, and behavior contracts.

#### Thresholds for Escalations and Placing Limits on Family Members

Thresholds for escalation varied greatly among respondents. For example, one medical director (respondent 4) described behavior contracts as useful whenever a family “deviated from expected behavior,” while one medical director (respondent 14) said they were useful only when “a disagreement is felt to harm the child,” and another (respondent 24) if behaviors are “threatening to staff or interfering with care.” Some respondents stated that their threshold for involving security and protective services teams was if family members exhibited threatening behavior, and one explicitly described these interventions as “last resorts” (respondent 13).

## Discussion

In this mixed-methods survey study of PICU physician and nursing leaders, we found that decision-making conflict monitoring, mediation, and mitigation strategies were used variably across institutions. We also found that a small majority of institutions have policies to help address conflict management, that few track conflicts and their outcomes, and even fewer track considerations of health equity. While most institutions have a DEI office, few involve them in conflict management. Leaders offered ideas for multipronged conflict prevention and response based on strategies implemented at their institutions and also highlighted needs and concerns with existing approaches. Learning from existing management approaches may help develop standardized, generalizable interventions to reduce conflict, improve interventions, and reduce subjectivity in the application of interventions.

Respondents shared a wide range of indications for utilizing different approaches. Although more than half of institutions surveyed had policies and procedures for conflict management, most reported they lack specific guidance for common types of conflict. Existing evidence suggests that protocolization helps reduce the impact of biases and subjectivity on decision-making in the hospital setting.^22,23,24,25,26^ When policies for challenging issues are developed in siloes, variability in approaches is substantial.^27^ Developing policies and procedures in a standardized manner across institutions may improve this variability. Critically, the shaping of policies and procedures ought to consider a broad range of goals, including seeking consensus on clear indications for specific interventions, reducing the harms of conflict escalations on families, and mitigating disparities in approaches,^28^ which may benefit from input from diverse interested groups.^28,29^ Cross-disciplinary approaches to conflict resolution and management may help to develop interventions for PICUs.^30,31^

Most respondents reported their institutions did not track conflict outcomes, including disparities in outcomes. Tracking and transparently reporting outcomes has been found to be beneficial for promoting a culture of safety and reducing incidence of medical errors and hospital-acquired conditions.^32,33,34^ Respondents at institutions that tracked behavior contracts were significantly more likely to use a more formal approach to conflict management, turning more frequently to less punitive interventions (eg, ethics consults)^10^ and less frequently to more potentially punitive and harmful interventions (eg, behavior contracts)^9,35^ with more objective criteria for initiating such interventions. Tracking and internally reporting conflict outcomes, including disparities in conflict incidence and management, might help teams be prepared for conflict and allow institutions to measure outcomes over time to better understand the root causes of conflicts and create tangible targets for intervention and improvement.

Regarding consultation with others, social work was reported to be utilized in nearly all conflicts. Security consultations and behavior contracts, often seen as punitive interventions,^8,35^ were reported as being used more frequently than ethics consultations.^10^ Given their training and role as a neutral team member, ethics consultants may be an underutilized support that may be well positioned for conflict mediation, through case-specific consultations, and conflict prevention, through regular meetings with clinical team members and ethicists.^10,36,37,38^ DEI committees and experts were very infrequently reported to be systematically utilized. Existing work indicates that intentional involvement of experts in health equity in conflicts and challenges faced by families may serve to help raise awareness of biases and mitigate their harms.^10,28,39^ Importantly, attention to staffing, training, and adequate support is needed to sustain current use of resources (such as social work) and to introduce potential increased use of resources (such as ethics consultation and DEI experts).

Given the high prevalence of decision-making conflict in the PICU, it is surprising that we found very few clinical team members received training in tools to prevent or reduce conflict and that nursing and social work teams received more training than physicians. Although physicians often lead team meetings and discussions when conflict occurs and are frequently tasked with responding to or mediating conflict, they may be the least equipped—and are certainly the least formally trained—to manage it. Existing evidence supports that health care professional education on deescalation, mediation, and communication improves parent and clinician satisfaction and may reduce conflict.^40^

While there are no previously published data of which we are aware about the overall frequency of behavior contract use in the inpatient setting in adults or pediatrics, existing work describes the potential harms of their use as well as disparities in their use.^9,35^ Behavior contracts are used more frequently for PICU patients (compared with other hospitalized patients), for patients identifying as Black (compared with those identifying as White), and for patients receiving end-of-life care.^41^ Security consults are utilized more for Black patients than for White patients.^8^ Given the potential harms and stigma associated with these interventions and their known disparate application in conflict resolution, it may be beneficial to test alternative strategies, such as transitioning to different behavior response interventions or implementing clear and generalizable indications for behavior contract use.^9,35^

Some respondents shared descriptions of multidisciplinary interventions to prevent and respond to conflict effectively. One example, called PEACE rounds, involved weekly meetings with ethicists and unit leadership to discuss challenging cases so that potential conflicts are caught early and can be addressed prior to escalation. PEACE rounds have been shown to positively impact factors contributing to moral distress and to possibly shorten patient lengths of stay.^21^ Given that moral distress was cited by respondents as a key reason for escalated conflicts, interventions that serve to address moral distress may help prevent conflict and escalation. Respondents spoke positively about implementing different strategies to set expectations on admission that are standard, reinforced, and clearly communicated. Existing work describes the potential utility of bidirectional, “mutually agreed upon,” “co-agreements,” that may avoid “external blame and one-sided enforcement of rules.”^31^ Another preventive process suggested by many respondents was the use of multidisciplinary teams and standardized decision support tools for common decisions. While such approaches exist in some institutions for tracheostomy decision-making,^42,43,44^ they are lacking for most other common shared PICU decisions,^45^ so future work would benefit from determining which common decisions may benefit from support tools, followed by development and implementation of supports.

### Limitations

Our study has several limitations. First, we surveyed only a sample of PICU physician and nursing leadership, and the sampling methodology was not random. We sought and achieved a representative sampling of different sizes and types of PICUs. We intentionally chose to survey PICU leadership, rather than institutional leadership, which allowed us to learn about conflict management at the division level, yet our findings are therefore limited to the institutional knowledge of those surveyed. Because we obtained PICU nursing leadership contacts from PICU physician leaders, we were limited in the total number we approached and undersampled nursing leadership. We analyzed responses in aggregate, so we were unable to compare results from nursing vs physician leaders. In addition, important voices are missing that would provide a comprehensive understanding of the current landscape, notably, patient families, social work, security, ethics, and patient-family relations representatives; future work is needed to capture these critical perspectives. Although our survey response rate was relatively high at 60% for medical leadership and 40% for nursing leadership, we were underpowered to assess differences across some of the domains.^46^ Furthermore, we are limited by social desirability and response bias.

## Conclusions

The results of our survey study indicate wide variability in currently used processes for conflict monitoring, prevention, mediation, and management. We found differences in what PICU physician and nursing leaders believe to be best practice. PICU size was not associated with this variability, but PICUs that monitored conflicts did practice conflict management significantly differently compared with those that did not monitor conflicts. Our findings indicate a need to develop standardized and evidence-based processes to ensure greater effectiveness by clinical teams and leaders in addressing conflict and reducing potential disparities in outcomes. Effective conflict management is unlikely to be a one-size-fits all approach, but rather to require using multipronged processes that implement ways to prevent conflict, along with a toolkit that can help mediate and manage different categories of conflicts. Understanding existing processes is a critical step toward developing strategies and standardizing interventions to reduce conflict and ultimately to improve management, outcomes, and health disparities related to conflict between parents and clinicians.

## Notes

Supplement 1. **eAppendix.** Survey Administered **eFigure 1.** Conflict Resource Use **eFigure 2.** Behavior Contract Use

Supplement 2.
Data Sharing Statement
