# Supplementary material for: Pediatric Intensive Care Unit Conflict Management Perspectives Among Physician and Nurse Leaders
Source: JAMA Netw Open. 2025 May 15;8(5):e259783. doi: 10.1001/jamanetworkopen.2025.9783 (PMC12082374; doi:10.1001/jamanetworkopen.2025.9783)
Supplement: Supplement 1. — eAppendix. Survey Administered eFigure 1. Conflict Resource Use eFigure 2. Behavior Contract Use [file jamanetwopen-e259783-s001.pdf]

Supplemental Online Content

Olszewski AE, Shah SK, Barrera L, et al. Pediatric intensive care unit conflict management perspectives among physician and nurse leaders. *JAMA Netw Open.* 2025;8(5):e259783. doi:10.1001/jamanetworkopen.2025.9783

eAppendix. Survey Administered

eFigure 1. Conflict Resource Use

eFigure 2. Behavior Contract Use

This supplemental material has been provided by the authors to give readers additional information about their work.

© 2025 Olszewski AE et al. *JAMA Network Open*.

eAppendix. Survey Administered

Qualtrics used for survey, with each respondent receiving a unique link.

In this survey, we are going to ask you about your experiences with conflict, conflict mediation, and behavior modification. We define conflict as dispute, disagreement, or difference of opinion related to the management of a patient in the ICU involving more than one individual and requiring some decision or action.
Your participation in this survey will be used to help understand existing institutional approaches, and their potential strengths and weakness, in order to improve the way in which institutions approach conflict.
Participation is voluntary, anonymous, and confidential. We are also attaching an information sheet indicating that you will be consenting to participate in the survey through completion of the survey – there is no other signed documentation necessary for consent. If you have any questions or would like any further information about the study, please feel free to contact me via email.

We plan to survey people in other multidisciplinary leadership positions about this topic as well. In order to do so, we'd like to ask you for the name and email address of the people who fill the following roles at your institution.
1. nursing leadership in the PICU
2. hospital ethics committee/clinical ethics consultant leadership
3. hospital security leadership
4. social work leadership in the PICU
5. hospital patient family experience or patient advocate leadership

Demographics
First, tell us about yourself and your role:
1. What is your age?
   a. < 25 years old
   b. 26 to 35 years old
   c. 36 to 45 years old
   d. 46 to 55 years old
   e. Older than 55 years old
2. What is your gender?
   a. Female
   b. Male
   c. Non-binary
   d. Other
3. How many years have you served in your current role?
   a. <5 years
   b. 6-10 years
   c. 11-15 years
   d. >16 years
4. What percentage of your time is spent in patient care?
   a. <25%
   b. 25-50%
   c. 51-75%
   d. >75%

© 2025 Olszewski AE et al. *JAMA Network Open*.

Next, tell us about your hospital:

1. How many beds are in your PICU?
    a. < 10
    b. 11-20
    c. 21-30
    d. 31-40
    e. > 40
    a. I don't know

2. In what geographic region is your hospital located?
    a. Northeast
    b. Midwest
    c. South
    d. West

3. Is your hospital urban or rural?
    a. urban
    b. rural

4. Is yours a teaching hospital?
    a. yes
    b. no

Behavior management strategies/PICU Leadership Questions

1. Does your institution have a procedure/policy for escalations in conflict or behavior modification?
    a. yes
    b. no
    c. I don't know

2. If so, please share it, and describe how it is typically used. Regardless of how you share it, we will anonymize the results. However, please feel free to share an anonymized version.

3. If not, please describe the process that is typically used.

For the remaining survey questions, please answer to the best of your knowledge.

1. What are common reasons for conflicts that escalate to leadership involvement, and how frequent would you estimate each to be?

	Never	Sometimes	Frequently
Poor communication			
Family problems comprehending prognosis			
Unavailability of parents			
Care plan disagreements			
Life-sustaining treatment issues			
Coping problems (e.g. anxiety, anger)			
Other			

© 2025 Olszewski AE et al. *JAMA Network Open*.

2. If you selected "other," or have thoughts on other common sources of conflict, please describe.

3. If and when conflict mediation techniques are employed with families and clinical teams, what types of conflict mediation techniques are used, and with what frequency?

	Never	Sometimes	Frequently
De-escalation			
Motivational interviewing			
Third party mediation (someone external to conflict)			
Other			

4. If you selected "other," or have thoughts on other conflict mediation approaches, please describe.

5. Which of the following conflict mediation techniques do the following team members receive training in? Please skip this question if you do not know the answer.

	Bedside nursing	Nursing leaders	Medical trainees	Frontline providers	Attending staff	Medical leaders
De-escalation						
Motivational interviewing						
Third party mediation (someone external to conflict)						

6. Do you track outcomes of conflicts, including disparities in outcomes?
   a. yes
   b. no

7. Do you have a diversity, equity, and inclusion group or office at your institution?
   a. Yes
   b. No

8. If yes, is this group involved in conflicts between families and clinical teams?
   a. Yes – in all conflicts
   b. Yes – in a systematic way for some conflicts
   c. Yes – in an ad hoc/as needed fashion
   d. No

9. When imposing limits on families in response to conflict, how often are each of the following types of limits imposed?

© 2025 Olszewski AE et al. *JAMA Network Open*.

	Never	Sometimes	Frequently
Behavior contracts			
Security involvement			
Social work involvement			
Ethics involvement			
Patient-family relations involvement			
Other			

10. For the escalation techniques marked “other,” please describe.

11. Are behavior contracts tracked at your institution?
    a. yes
    b. no
    c. I don’t know

12. When behavior contracts are used, how frequently are they used for the following indications:

	Never	Sometimes	Frequently
Physical aggression			
Verbal aggression			
Both physical and verbal aggression			
Audio/video recording			
Breach of COVID rules			
Domestic violence/disturbance			
Obstruction of care			
Non-adherence to hospital policies (i.e. exhibiting racism, sexism, intolerance, threatening)			
Other			

For the behavior contract reasons marked “other,” please describe.

13. Are disparities and equity considerations tracked among behavior contracts?
    a. yes
    b. no
14. If so, are these reviewed, or is this tracking used in some way?
    a. yes
    b. no

Conflict scenarios

<u>First Conflict</u>

1. A 12-month-old child has severe hypoxic ischemic encephalopathy from birth asphyxia. They require invasive mechanical ventilator support and are in the intensive care unit. They are profoundly neurologically depressed, but grimace to painful stimuli. The family is requesting tracheostomy and G-tube placement, and the clinical team is concerned that these procedures are futile. Tell us in your own words how you generally approach cases like this.

© 2025 Olszewski AE et al. *JAMA Network Open*.

2. Would you support the family's request for intervention, or decline to provide the interventions?
    a. Support family’s request
    b. Decline family’s request
    c. Other
3. If you selected “Other,” please describe.
4. Now, please choose from *one* of the following approaches as an initial approach.

De-escalation
Motivational interviewing
Third party mediation
Behavior contracts
Security involvement
Social work involvement
Nursing leadership involvement
Ethics involvement
Patient-family relations involvement
Family care conference
Provider only conference
Other

5. If you selected “Other,” please describe.
6. Why would your team select this initial approach?
7. Please indicate *any* other choices that you might consider helpful in case like this one.

De-escalation
Motivational interviewing
Third party mediation
Behavior contracts
Security involvement
Social work involvement
Nursing leadership involvement
Ethics involvement
Patient-family relations involvement
Family care conference
Provider only conference
Other

8. If you selected “Other,” please describe.
9. Does your hospital or PICU have a formal policy for scenarios like this specifically?

<u>Second Conflict</u>

1. For the same patient, consider some additional factors - now, the clinical team is concerned that the life-sustaining treatments being provided are “futile,” because the patient “has no future.” The clinician is concerned that that the child is “suffering,” that the family “doesn’t get it,” and is “in denial.” Further, the team feels frustrated that the family is rarely at the bedside, giving them a limited view of the patient’s prognosis. Tell us in your own words how you generally approach cases like this.
2. Now, please choose from *one* of the following approaches as an initial approach.

De-escalation
Motivational interviewing
Third party mediation
Behavior contracts

© 2025 Olszewski AE et al. *JAMA Network Open*.

Security involvement
Social work involvement
Nursing leadership involvement
Ethics involvement
Patient-family relations involvement
Family care conference
Provider only conference
Other

3. If you selected “Other,” please describe.
4. Why would your team select this initial approach?
5. Please indicate *any* other choices that you might consider helpful in case like this one.

De-escalation
Motivational interviewing
Third party mediation
Behavior contracts
Security involvement
Social work involvement
Nursing leadership involvement
Ethics involvement
Patient-family relations involvement
Family care conference
Provider only conference
Other

6. If you selected “Other,” please describe.
7. Does your hospital or PICU have a formal policy for scenarios like this specifically?

Third Conflict

1. A patient with tracheostomy and ventilator dependence is admitted with escalated ventilator settings in the setting of a pneumonia. One day, the patient’s father raises his voice at the bedside nurse, because the manner in which she suctioned was not how they do things at home. The family now “refuses to attend rounds,” and is frequently described as “escalated,” and “angry.” The nurse expresses that they feel unsafe with the patient’s father. Tell us in your own words how you generally approach cases like this.
2. Now, please choose from *one* of the following approaches as an initial approach.

De-escalation
Motivational interviewing
Third party mediation
Behavior contracts
Security involvement
Social work involvement
Nursing leadership involvement
Ethics involvement
Patient-family relations involvement
Family care conference
Provider only conference
Other

3. If you selected “Other,” please describe.
4. Why would your team select this initial approach?

© 2025 Olszewski AE et al. *JAMA Network Open*.

5. Please indicate *any* other choices that you might consider helpful in case like this one.

De-escalation
Motivational interviewing
Third party mediation
Behavior contracts
Security involvement
Social work involvement
Nursing leadership involvement
Ethics involvement
Patient-family relations involvement
Family care conference
Provider only conference
Other

6. If you selected "Other," please describe.
7. Does your hospital or PICU have a formal policy for scenarios like this specifically?

<u>Fourth Conflict</u>

1. A 14-year-old is admitted to the PICU with multi-organ dysfunction and altered mental status in the setting of severe restrictive eating disorder. The family refuses NG placement for feeding. After several days, her organ function is mildly improved, and the team again strongly recommends NG placement. The patient and her parents refuse, requesting that IV nutrition be completed and she be discharged home, as they believe she will do best at home. Tell us in your own words how you generally approach cases like this.
2. Would you support the family's request, or proceed with NG placement and admission?
    a. Support family's request
    b. Decline family's request
    c. Other
3. If you selected "Other," please describe.
4. Now, please choose from *one* of the following approaches as an initial approach.

De-escalation
Motivational interviewing
Third party mediation
Behavior contracts
Security involvement
Social work involvement
Nursing leadership involvement
Ethics involvement
Patient-family relations involvement
Family care conference
Provider only conference
Other

5. If you selected "Other," please describe.
6. Why would your team select this initial approach?
7. Please indicate *any* other choices that you might consider helpful in case like this one.

De-escalation
Motivational interviewing
Third party mediation
Behavior contracts

© 2025 Olszewski AE et al. *JAMA Network Open*.

Security involvement
Social work involvement
Nursing leadership involvement
Ethics involvement
Patient-family relations involvement
Family care conference
Provider only conference
Other

8. If you selected “Other,” please describe.
9. Does your hospital or PICU have a formal policy for scenarios like this specifically?

© 2025 Olszewski AE et al. *JAMA Network Open*.

eFigure 1. Conflict Resource Use

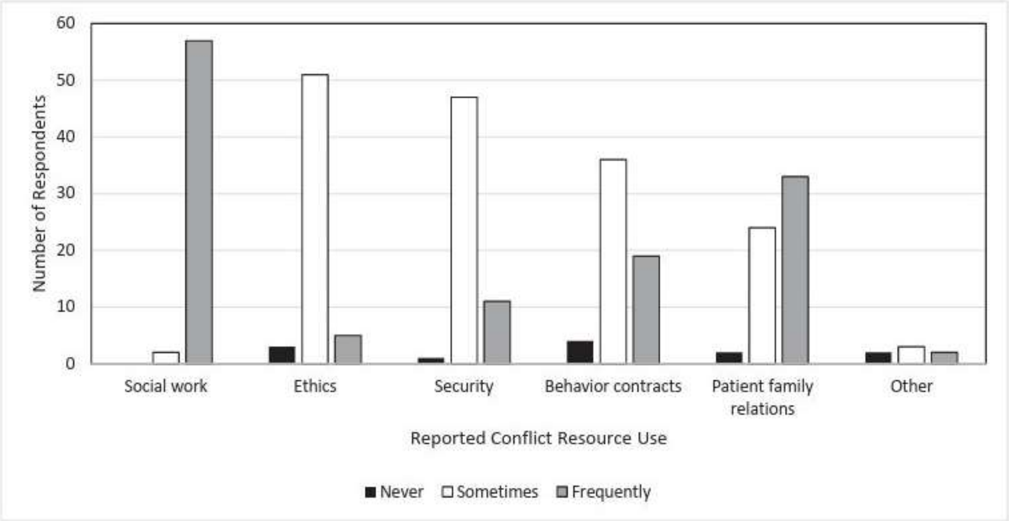

Respondents answered whether the listed conflict resources were used frequently, sometimes, or never (n = 59).

© 2025 Olszewski AE et al. *JAMA Network Open*.

eFigure 2. Behavior Contract Use

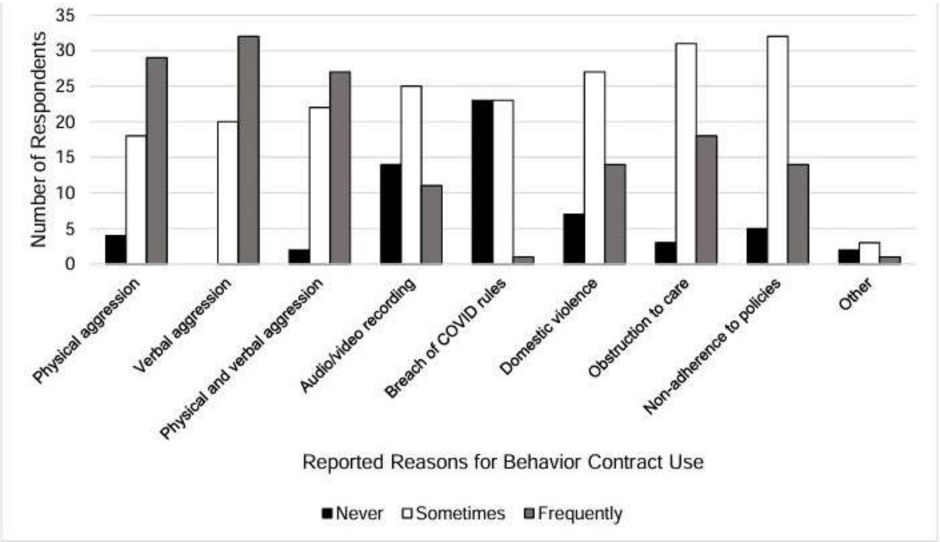

Respondents shared whether the listed reasons for behavior contracts were applied frequently, sometimes, or never (n = 52).

© 2025 Olszewski AE et al. *JAMA Network Open*.
