# Supplementary material for: Pediatric Intensive Care Unit Conflict Management Perspectives Among Physician and Nurse Leaders
Source: JAMA Netw Open. 2025 May 15;8(5):e259783. doi: 10.1001/jamanetworkopen.2025.9783 (PMC12082374; doi:10.1001/jamanetworkopen.2025.9783)
Supplement: Supplement 2. — Data Sharing Statement [file jamanetwopen-e259783-s002.pdf]

Data Sharing Statement

Olszewski. Pediatric Intensive Care Unit Conflict Management Perspectives Among Physician and Nurse Leaders. *JAMA Netw Open*. Published May 15, 2025.
doi:10.1001/jamanetworkopen.2025.9783

Data
Data available: Yes
Data types: Data dictionary
How to access data: olszewskia@upmc.edu
When available: With publication

Supporting Documents
Document types: Informed consent form
How to access documents: olszewskia@upmc.edu
When available: With publication

Additional Information
Who can access the data: anyone requesting
Types of analyses: for any purpose
Mechanisms of data availability: with investigator support
Any additional restrictions: none
